# Correlation between sleep duration and prevalence of hypertension: the China Health and Nutrition Survey

**DOI:** 10.1590/1414-431X2024e13868

**Published:** 2024-11-25

**Authors:** Hong-Shan Guan, Hai-Juan Shangguan

**Affiliations:** 1 Department of Cardiology, Renmin Hospital of Wuhan University, Wuhan, Hubei, China; 2 Department of Cardiology, Wuhan Aisa Heart Hospital, Wuhan, Hubei, China

**Keywords:** Sleep duration, Hypertension, Epidemiology

## Abstract

It is increasingly thought that sleep is a lifestyle factor that contributes to hypertension. However, the correlation between sleep duration and hypertension in the Chinese population remains largely unexplored. This study utilized data from the 2009 China Health and Nutrition Survey to investigate the correlation between sleep duration and hypertension. Average hours of sleep per day were grouped into following categories: ≤6, 7-9, and ≥10 h. The frequency of hypertension and odds ratio (OR) were computed across different sleep duration categories. Individuals sleeping 7-9 h per day were designated as the control group. Logistic regression was utilized for multivariate analysis. Among the 9435 participants, the mean sleep duration was 7.9±1.2 h. The prevalence of hypertension was 34.1, 21.7, and 29.3% for individuals sleeping ≤6, 7-9, and ≥10 h per day, respectively. Following adjustments for age, gender, body mass index, and diabetes, a significant association was observed between prolonged (≥10 h) sleep duration and hypertension. Compared to those sleeping 7-9 h per day, the OR for hypertension was 1.21 (95%CI: 1.02-1.43, P=0.03) for individuals sleeping ≥10 h per day. This study suggested that sleeping ≥10 h per day is associated with a higher risk of hypertension in adults.

## Introduction

The prevalence of hypertension in China has seen a notable increase over the past few decades, from 11.3% in 1991 to 23.2% in 2015 (1,2). An estimated 244.5 million Chinese are affected by hypertension (2). The aging population and the increasing prevalence of risk factors will lead to a growing burden of hypertension (3,4). Projected trends indicate that the population affected by hypertension will increase in the coming decades (5). Thus, it is crucial to identify the risk factors associated with hypertension in order to avoid its occurrence and subsequently decrease the impact of related illnesses.

Increasing evidence indicates that the duration of sleep could serve as a significant predictor of an individual's health status (6), and that there is an association between both long and short sleep duration and a range of negative health consequences, such as cardiovascular illnesses and all-cause mortality (7-11). Sleeping duration has become a novel modifiable treatment target for prevention and treatment of many chronic diseases (12-16). Recent research has revealed that insufficient sleep is a risk factor for hypertension, indicating the possible detrimental effect of poor sleep on the development of high blood pressure (16-19). Most studies associating sleep duration with hypertension include only limited confounders for hypertension and are inadequate to describe the full picture of the association between sleep and hypertension. Furthermore, it is still uncertain whether there is a correlation between the length of sleep and hypertension and other metabolic indicators in the Chinese population.

Therefore, we conducted a study to investigate the correlations between the amount of sleep and hypertension by analyzing data from the China Health and Nutrition Survey (CHNS). Our hypothesis was that both shorter and longer sleep duration would be related to a higher prevalence of hypertension compared to medium duration.

## Material and Methods

### Study population

The CHNS is an ongoing multi-wave longitudinal survey that started in 1989 (20). A total of 228 communities were selected from 9 provinces, namely Guangxi, Guizhou, Heilongjiang, Henan, Hubei, Hunan, Jiangsu, Liaoning, and Shandong, utilizing a multistage, random cluster approach. Surveys were conducted every 2-4 years, with a total of 9 waves between 1989 and 2011. The 2009 CHNS survey marked the first blood sample collection, enabling the availability of fasting blood data for participants aged 7 years and older. The current study used data from the 2009 wave using a cross-sectional design. Additional details regarding the CHNS procedures are provided elsewhere (20).

### Sleep duration

The duration of sleep was ascertained by in-person interviews using the following query: “On average, how many hours do you typically sleep per day, both daytime and nighttime?”. Then, we further grouped participants by the sleep duration into 3 categories: short (≤6 h), normal (7-9 h), and long (≥10 h) (21).

### Hypertension assessment

Blood pressure (BP) measurements were obtained in triplicate by experienced physicians following a 10-min seated rest, utilizing a mercury sphygmomanometer and adhering to standard protocol. The mean value of the three BP measurements was used in the analysis. Hypertension was defined as either currently taking antihypertensive drugs or having a mean systolic blood pressure (SBP) ≥140 mm Hg or mean diastolic blood pressure (DBP) ≥90 mm Hg.

### Covariates

Potential confounders used for adjustment included age, educational levels, occupation, smoking, drinking, body mass index, and diabetes. Sex, year of birth, and age were self-reported in the face-to-face survey. Weight was determined with precision to the nearest 0.1 kg with subjects dressed in lightweight garments, using a calibrated beam scale. The height was determined with a handheld stadiometer, without shoes, with a precision of 0.2 cm. The body mass index (BMI) was calculated by dividing the weight in kilograms by the square root of the person's height in meters.

Following a minimum of 8 h of fasting throughout the night, blood samples of 12 mL were obtained from participants aged 7 years and above. Subsequently, the whole blood was promptly centrifuged, and the serum was immediately tested for glucose and hemoglobin A1c (HbA1c). Following this, plasma and serum samples were cryopreserved at a temperature of -86°C for later laboratory analysis. All samples underwent analysis at a national central laboratory the Cardiovascular Institute and Fuwai Hospital in Beijing, China, with stringent quality control measures in place. Glucose levels were determined using the glucose oxidase-phenol and aminophenazone method on a Hitachi 7600 analyzer (Hitachi; Japan), while serum insulin levels were assessed using the radioimmunoassay method on a XH-6020 gamma counter (North Institute of Biological Technology, China). Homeostatic Model Assessment of Insulin Resistance (HOMA-IR) was calculated using the formula: insulin × glucose / 6.945 pmol/L. Diabetes was defined according to the 2010 American Diabetes Association criteria, including fasting plasma glucose (FPG) levels ≥7.0 mmol/L, HbA1c levels ≥6.5%, or the use of glucose-lowering medications (22).

### Statistical analysis

Demographic, anthropometric, and hypertension data were summarized by sex. Categorical data are reported as percentages, whilst continuous variables are reported as means±SD. Because of the skewed distribution, insulin data are reported as median and lower and upper quartiles. Comparisons were performed using *t*-tests and χ^2^ tests for continuous and categorical data, respectively. The Mann-Whitney U test was used specifically for comparing insulin levels. The study utilized binary logistic regression models to investigate the correlation between sleep duration and hypertension. Adjusted ORs along with their corresponding 95% confidence intervals (CIs) are reported. Statistical analyses were conducted using SAS (USA), and significance was determined by a two-tailed P value <0.05.

## Results

### Characteristics of the study subjects

This study comprised a total of 9435 adults aged 18 years and above. Table 1 presents the demographic and clinical attributes of the individuals involved in the investigation, categorized by gender. The mean age of the subjects was 50.5±15.4 years and 47.3% of them were men. The average sleep duration was 8.0±1.2 h and 7.9±1.2 h in men and women, respectively. Overall, 10.2, 78.6, and 11.2% of participants reported sleeping ≤6, 7-9, and ≥10 h per day, respectively.

**Table 1:** Characteristics of study participants by gender.

Characteristics	Men	Women	Total
n	4463	4972	9435
Age (years)	50.3±15.3	50.6±15.4	50.5±15.4
Education			
<7 years	1425 (31.5)	2455 (48.8)	3880 (40.6)
7-9 years	1771 (39.2)	1520 (30.2)	3291 (34.4)
10-12 years	632 (14.0)	487 (9.7)	1119 (11.7)
≥12 years	695 (15.4)	574 (11.4)	1269 (13.3)
Occupation			
No job/retired	1477 (32.7)	2505 (49.7)	3982 (41.7)
Blue collar	538 (11.9)	411 (8.2)	949 (9.9)
White collar	2508 (55.5)	2120 (42.1)	4628 (48.4)
Current smoking	2437 (54.6)	180 (3.6)	2649 (28.1)
Current drinking	2652 (59.4)	445 (9.0)	3097 (32.8)
Sleep duration (h)	8.0±1.2	7.9±1.2	7.9±1.2
Diabetes	117 (3.1)	131 (2.6)	268 (2.8)
Hypertension	1082 (24.2)	1168 (23.5)	2250 (23.9)
BMI (kg/m^2^)	23.3±3.4	23.4±3.5	23.4±3.5
SBP (mm Hg)	126±18	124±20	125±19
DBP (mm Hg)	82±11	79±11	81±11
TC (mmol/L)	4.8±1.0	4.9±1.0	4.9±1.0
LDL-C (mmol/L)	2.9±1.0	3.0±1.0	3.0±1.0
HDL-C (mmol/L)	1.4±0.5	1.5±0.5	1.4±0.5
TG (mmol/L)	1.8±1.7	1.6±1.2	1.7±1.5
Glucose (mmol/L)	5.5±1.6	5.3±1.3	5.4±1.5

Data are reported as mean and SD or number and percentage. BMI: body mass index; SBP: systolic blood pressure; DBP: diastolic blood pressure; TC: total cholesterol; LDL-C: low-density lipoprotein cholesterol; HDL-C: high-density lipoprotein cholesterol; TG: triglyceride.

### Incidence of hypertension among different sleep duration categories

**Figure 1:**
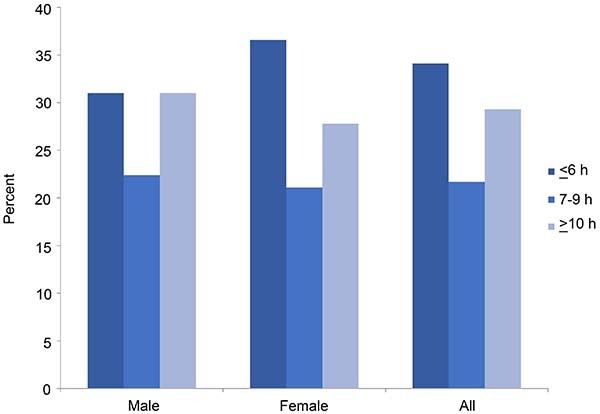
Prevalence of hypertension (%) by gender by sleep duration categories.

Figure 1 illustrates the occurrence of hypertension across different sleep duration categories, categorized by gender, among all 9435 participants. Notable variations in the prevalence of hypertension were noted among male and female participants. Those who slept 7-9 h per day exhibited the lowest prevalence of hypertension. The incidence of hypertension was 34.1, 21.7, and 29.3% for individuals who slept 6 h or less, 7-9, and 10 h or more each day, respectively (Table 2). Male, female, and overall participants who slept 7-9 h per day had the lowest mean levels of SBP.

**Table 2:** Blood pressure (mean ± SD) and prevalence of hypertension (%, n) across different categories of sleep duration in participants.

Characteristics	Sleep duration		
	≤6 h	7-9 h	≥10 h
Men (n=4463)			
SBP	129±19	125±17	128±20
DBP	83±12	82±11	82±12
Hypertension	30.1 (131/423)	22.4 (790/3520)	30.1 (161/520)
Women (n=4972)			
SBP	132±22	122±19	125±22
DBP	82±12	79±11	79±12
Hypertension	36.6 (191/522)	21.1 (816/3871)	27.8 (161/579)
All (n=9435)			
SBP	130±21	124±18	126±21
DBP	83±12	80±11	80±12
Hypertension	34.1 (322/945)	21.7 (1606/7391)	29.3 (322/1099)

SBP: systolic blood pressure; DBP: diastolic blood pressure.

### Relationship between duration of sleep and hypertension

Table 3 displays the unadjusted and adjusted odds ratios (ORs) and 95% confidence intervals (CIs) for sleep duration category and hypertension among all 9435 participants. After the adjustment for age, educational levels, occupation, smoking, drinking, BMI, and diabetes, a significant association was observed between prolonged (≥10 h) sleep duration and hypertension. Compared to those sleeping 7-9 h per day, OR for hypertension was 1.21 (95%CI: 1.02-1.43, P=0.03) in individuals sleeping ≥10 h per day.

**Table 3:** Association between sleep duration categories and prevalence of hypertension by gender.

	Sleep duration		
	<7 h	7-9 h	>9 h
Men (n=4463)			
Case/total study samples	131/423	790/3520	161/520
*Model 1, OR (95%CI), P value	1.55 (1.24, 1.93), <0.001	1.00 (ref)	1.55 (1.27, 1.90), <0.001
*Model 2, OR (95%CI), P value	1.11 (0.87, 1.42), 0.411	1.00 (ref)	1.33 (1.05, 1.69), 0.016
Women (n=4972)			
Case/ total study samples	191/522	816/3871	161/579
*Model 1, OR (95%CI), P value	2.16 (1.78, 2.62), <0.001	1.00 (ref)	1.44 (1.18, 1.76), <0.001
*Model 2, OR (95%CI), P value	1.20 (0.96, 1.51), 0.115	1.00 (ref)	1.09 (0.86, 1.39), 0.471
All (n=9435)			
Case/total study samples	322/945	1606/7391	322/1099
*Model 1, OR (95%CI), P value	1.86 (1.61, 2.15), <0.001	1.00 (ref)	1.49 (1.30,1.72), <0.001
*Model 2, OR (95%CI), P value	1.15 (0.98, 1.36), 0.096	1.00 (ref)	1.21 (1.02,1.43), 0.027

CI: confidence interval; OR: odds ratio. *Model 1 was unadjusted; Model 2 was adjusted for age, educational levels, occupation, smoking, drinking, body mass index, and diabetes.

## Discussion

In this cross-sectional study of 9435 community-based participants from CHNS, we found that prolonged sleep duration (≥10 h) was associated with a higher risk of hypertension in adults, even after adjusting for age, educational levels, occupation, smoking, drinking, body mass index, and diabetes. Moreover, this association was more obvious in men. These findings add important information to the results from several previous large population-based studies (23-28).

Numerous population-based studies have explored the correlation between sleep duration and hypertension, and have been inconsistent (27-29). Through a meta-analysis of 9 populations, Wang et al. (30) found that individuals who sleep for either short or long durations are more likely to develop hypertension. These associations are driven by distinct mechanisms. This recent meta-analysis using a dose-response model proposed a U-shaped association between sleep duration and risk of hypertension. It indicated that individuals with a sleep duration of 7-8 h per day had the lowest risk of hypertension (30). However, no significant association was detected in those self-reporting ≤6 h, although prevalence of hypertension was higher in those with short sleep duration. The smaller sample size of the short sleep duration group might have affected the statistical power of the analysis, making it harder to observe significant associations.

Previous studies using objective data showed that short sleep duration and sleep duration variability were not associated with hypertension (31,32). In contrast, our study using subjective sleep duration data found that prolonged sleep duration was associated with higher risk for hypertension. This discrepancy may also be explained by the different methods for measuring sleep duration.

Despite individuals who slept for 7-9 h per day exhibiting the lowest prevalence of hypertension, following adjustments for age, gender, BMI, and diabetes, a significant association was observed between prolonged (≥10 h) sleep duration and hypertension.

The specific biological processes that explain the previously observed link between either insufficient or excessive sleep and the likelihood of developing hypertension are still not well understood. Insufficient sleep may influence metabolism and physiological function (33,34). Sleep restriction has been demonstrated to increase sympathetic nervous system activity (35), which could impact insulin sensitivity. Elevated glucose levels and insufficient insulin could both contribute to the development of insulin resistance and hypertension. In our study, both short and long self-reported sleep durations showed significant univariate associations with the prevalence of hypertension. However, once age, BMI, and diabetes were included in the multivariable model, the association between hypertension prevalence and short sleep duration became non-significant. This finding revealed that short sleep duration may not be a risk factor for hypertension per se but is confounded by age, BMI and lifestyle factors. Given that both obesity and hypertension are linked to sleep duration and serve as risk factors for hypertension, investigating the potential mediating effect of obesity and hypertension could aid in the understanding of the pathway from sleep disturbances to hypertension.

The current study had several limitations that deserve attention. First, sleep duration was self-reported by participants, and the reliability of this self-reported data was not validated in our study. Subjective reports of sleep duration can be influenced by individual perception differences, memory errors, and reporting biases (36-38). Therefore, potential misclassification of exposure might exist in our study. Second, cross-sectional data from CHNS was used in the analysis. The temporal relationship between sleep duration and hypertension-related metabolic markers cannot be established. Third, although known confounders like gender and age were included in the multivariable model, the probability of residual confounding could not be definitively excluded.

In summary, the results presented here demonstrated that prolonged sleep duration was associated with a higher risk of hypertension in adults, even after adjusting for age, educational levels, occupation, smoking, drinking, BMI, and diabetes. The results suggested that prolonged sleep may serve as a risk factor for hypertension, persisting even after accounting for various potential confounding factors. This highlights the need for further exploration into the intricate relationship between sleep and health to devise more effective preventive and intervention measures. Additionally, the findings offer crucial insights for healthcare providers and public health policymakers to better understand and manage one of the risk factors for hypertension.
